# The challenges of colposcopy for cervical cancer screening in LMICs and solutions by artificial intelligence

**DOI:** 10.1186/s12916-020-01613-x

**Published:** 2020-06-03

**Authors:** Peng Xue, Man Tat Alexander Ng, Youlin Qiao

**Affiliations:** 1 National Cancer Center/National Clinical Research Center for Cancer, Cancer Hospital, Chinese Academy of Medical Sciences and Peking Union Medical College, Beijing, China; 2 Department of Epidemiology and Biostatistics, School of Public Health, Chinese Academy of Medical Sciences and Peking Union Medical College, Beijing, China; 3 Tencent Healthcare, Shenzhen, China; 4 Affiliated Cancer Hospital of Zhengzhou University, Henan Cancer Hospital, Zhengzhou, China

**Keywords:** Artificial intelligence, Colposcopy diagnosis, Cervical cancer screening, Global elimination of cervical cancer

## Abstract

**Background:**

The World Health Organization (WHO) called for global action towards the elimination of cervical cancer. One of the main strategies is to screen 70% of women at the age between 35 and 45 years and 90% of women managed appropriately by 2030. So far, approximately 85% of cervical cancers occur in low- and middle-income countries (LMICs). The colposcopy-guided biopsy is crucial for detecting cervical intraepithelial neoplasia (CIN) and becomes the main bottleneck limiting screening performance. Unprecedented advances in artificial intelligence (AI) enable the synergy of deep learning and digital colposcopy, which offers opportunities for automatic image-based diagnosis. To this end, we discuss the main challenges of traditional colposcopy and the solutions applying AI-guided digital colposcopy as an auxiliary diagnostic tool in low- and middle- income countries (LMICs).

**Main body:**

Existing challenges for the application of colposcopy in LMICs include strong dependence on the subjective experience of operators, substantial inter- and intra-operator variabilities, shortage of experienced colposcopists, consummate colposcopy training courses, and uniform diagnostic standard and strict quality control that are hard to be followed by colposcopists with limited diagnostic ability, resulting in discrepant reporting and documentation of colposcopy impressions. Organized colposcopy training courses should be viewed as an effective way to enhance the diagnostic ability of colposcopists, but implementing these courses in practice may not always be feasible to improve the overall diagnostic performance in a short period of time. Fortunately, AI has the potential to address colposcopic bottleneck, which could assist colposcopists in colposcopy imaging judgment, detection of underlying CINs, and guidance of biopsy sites. The automated workflow of colposcopy examination could create a novel cervical cancer screening model, reduce potentially false negatives and false positives, and improve the accuracy of colposcopy diagnosis and cervical biopsy.

**Conclusion:**

We believe that a practical and accurate AI-guided digital colposcopy has the potential to strengthen the diagnostic ability in guiding cervical biopsy, thereby improves cervical cancer screening performance in LMICs and accelerates the process of global cervical cancer elimination eventually.

## Background

The burden of cervical cancer remains an important indicator of global health inequality. There are estimated 569,000 new cases and 310,000 deaths annually, among which approximately 85% occurred in low- and middle-income countries (LMICs) [1]. In 2018, the World Health Organization (WHO) called for global action towards the elimination of cervical cancer; one of the main strategies is to screen 70% of women between the age of 35 and 45 years and 90% of women managed appropriately by 2030, in order to achieve less than four new cases per 100,000 women [2]. In high-income countries, the occurrence of cervical cancer has significantly decreased by well-organized screening programs, which require infrastructure and extensively experienced clinicians which are lacking in LMICs [3, 4]. Such a decrease can be attributed to the effective prevention of cervical intraepithelial neoplasia (CIN) through primary screening, colposcopy, and treatment [5]. In conjunction with primary screening and treatment, traditional colposcopy plays an important role in guiding cervical biopsy but has become the main bottleneck limiting the screening performance in LMICs. Fortunately, artificial intelligence (AI), particularly deep learning, has been widely used for image-based subjective diagnosis in healthcare services [6, 7]. The digital colposcopy with the ability of high-definition imaging, in combination with deep learning, can offer opportunities for automatic image-based diagnosis. Development and application of AI-guided digital colposcopy has substantial potential to solve the colposcopic bottleneck and improve the performance of colposcopy and cervical cancer screening. To this end, we discuss the main challenges of traditional colposcopy and the solutions applying AI-guided digital colposcopy as an auxiliary diagnostic tool in LMICs.

## The challenges of diagnostic performance of colposcopy in LMICs

In LMICs, colposcopy is widely used to detect CINs and guide cervical biopsy sites for women who had abnormal cytology, human papillomavirus (HPV) infections, and clinical symptoms of suspected cervical diseases [8, 9]. Though colposcopy has made tremendous contributions to the prevention of cervical cancer in history, the overall performance of colposcopy remains unsatisfactory [10]. The diagnostic accuracy for cervical biopsy to detect CINs is reported to be relatively low, ranging from 30 to 70%, especially in LMICs due to a shortage of colposcopic service capability [11]. There are six challenges that may contribute to the unsatisfactory accuracy of colposcopy examination in LMICs.

First, the diagnostic performance of colposcopy strongly depends upon the subjective experience of operators, which requires the operators to recognize, process, and compare the perceived changes of acetowhitening epithelium (thickness, color, border irregularity, surface smoothness, the timing of appearing and fading, etc.) with the known features/criteria. The colposcopy not only focuses on cervical imaging technique, but also involves chemical staining of cervical epithelial cell which is influenced by solution quality, operation, and observation ways; thus, there are substantial inter- and intra-operator variabilities in the agreement of colposcopic impressions and pathology, ranging from 52 to 66% [12–14].

Second, due to the lack of experienced colposcopists in LMICs, the workload increases along with the expanded cervical cancer screening programs, exacerbating the diagnostic inaccuracy of colposcopy. Third, organizing colposcopy training courses should be viewed as an effective way to enhance the diagnostic ability of colposcopists, but implementing these courses in practice may not always be feasible to improve the overall diagnostic performance in a short period of time and may be difficult to scale across geographies. Besides, as the occurrence of cervical cancer is expected to decrease with HPV-vaccinated women entering the screening population, junior colposcopists will find it difficult to see many cases of high-grade cervical lesions or even cancer in clinical practice, which limits their accumulation of expertise and experience. Forth, although uniform diagnostic standards and strict quality control for colposcopy practice are released by relevant official organizations, many colposcopic practitioners due to the limited diagnostic ability and lacking professional training from LMICs are having a hard time to follow standardized recommendations to practice colposcopy examination, resulting in discrepant reporting and documentation of colposcopy impressions. Finally, the diagnostic performance of colposcopy may be adversely affected by changes in screening modality from cytology, cytology-HPV co-testing to primary HPV screening, since cervical lesions related to HPV infections are likely to be mild and harder to be identified than cytological abnormalities. In general, diagnosing cervical abnormalities is a great challenge for the colposcopists in LMICs.

## The solutions to development and application of AI-guided digital colposcopy

In LMICs like China, although convincing achievements have been achieved for cervical cancer prevention and control, many challenges still exist and need more innovative solutions [15]. The small subset of screened women with a possible high risk of CINs from a large population warrants referral to colposcopy for further clinical evaluation. However, the variable diagnostic performance frequently results in the suggestion of repeat colposcopy examination or interpretation as potentially false-negative findings, which pose a considerable burden for affected women. Women in LMICs continued to suffer the challenges of inadequate performance of traditional colposcopy in an already overburdened healthcare system. Researchers endeavor to find effective solutions to improve the performance of colposcopy and cervical cancer screening.

**Fig. 1:**
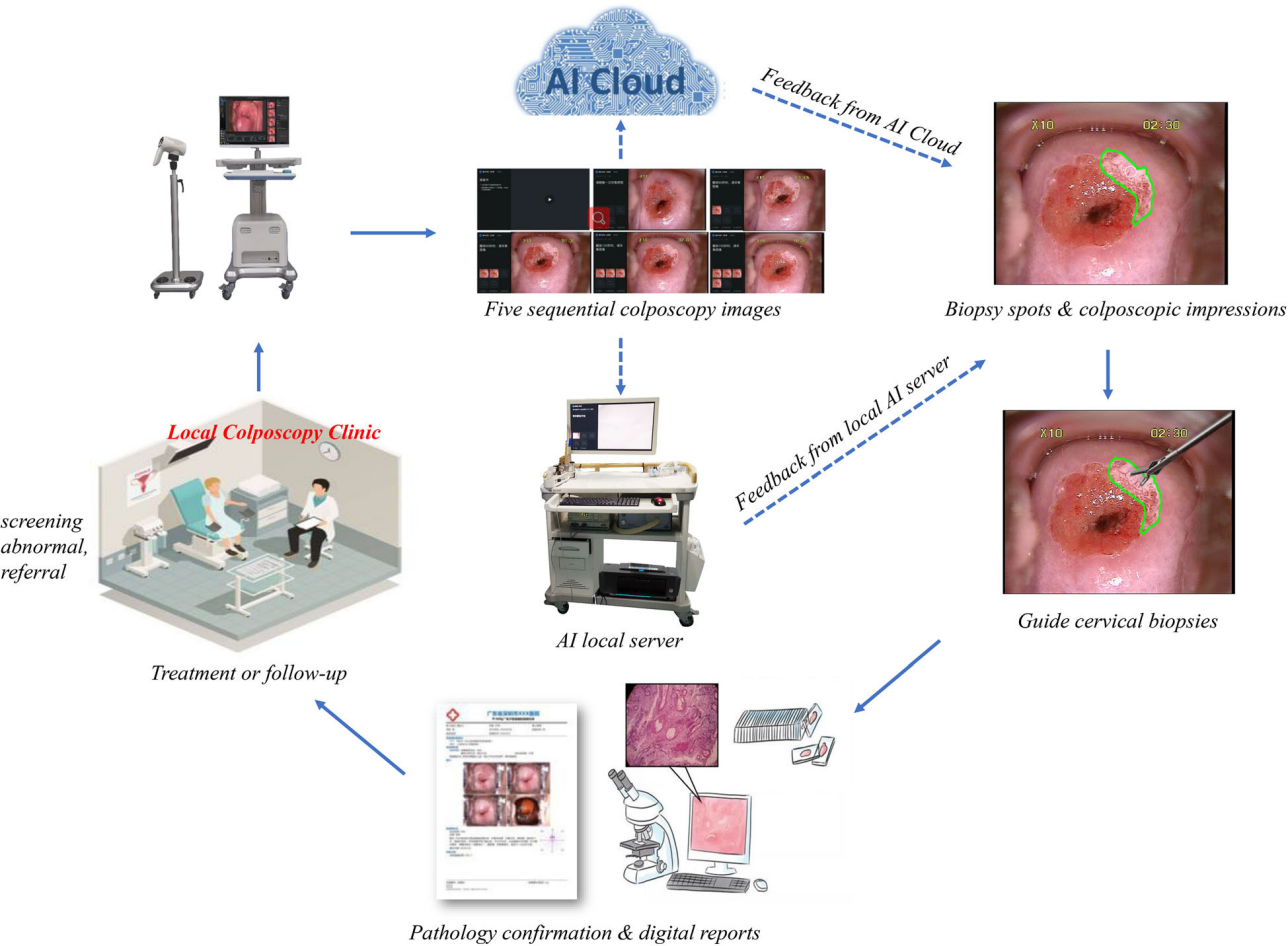
The diagnostic workflow of colposcopy clinic based on AI-guided digital colposcopy. Note: with abnormal screening results following cytology or HPV testing, women are generally referred to colposcopy clinic for AI-guided digital colposcopy evaluation, including biopsy spots as shown in green outline, and possibilities of cervical lesions. And the diagnostic results are later confirmed by pathology for the decision of clinical management (either immediate treatment or follow-up). During colposcopy examination, five sequential colposcopy images are captured and transmitted to one of two available clinical applications: (1) AI local server that is suitable for areas with poor network conditions and (2) AI Cloud that is for areas with good internet access. Both can provide a real-time response as an auxiliary diagnostic tool for colposcopists after they uploaded their colposcopic images to AI local server or the cloud platform. It also represents a useful training tool for new colposcopists. This Figure was created by the authors

As health systems and hospitals are more digitally enabled, traditional binocular colposcopy is also moving towards digital colposcopy, enabling colposcopists to access high-definition cervical images for diagnostic analyses. Although digital colposcopy has increased the accuracy of cervical examinations, it remains suboptimal in clinical practice due to a high degree of inter- and intra-colposcopist variability. Recent advances in AI can provide solutions to overcome these unsolved colposcopic bottlenecks. The AI methods, such as the deep learning-based algorithms, can learn features of cervical lesions from annotated colposcopy images which can then be integrated into the digital for automated colposcopy. This approach can address the diagnostic subjectivity of traditional colposcopy and improve the objectivity and performance of colposcopy with dynamic digital imaging. Another benefit offered by AI is that it could report diagnostic results in real time; therefore, the integration of AI algorithm into colposcopy equipment could help colposcopists to improve clinical workflow in a busy colposcopy clinic. In addition, due to the imbalance of medical resources in urban and rural areas of LMICs, the availability of a cloud-based AI platform could narrow the gap in colposcopy diagnosis between tertiary hospitals and primary care hospitals. Therefore, we believe that the development and application of AI-guided digital colposcopy have an important role in optimizing colposcopy services and augmenting clinical imaging judgment and is expected to improve the diagnostic performance of colposcopy through the detection of CINs and guidance of biopsy sites. At the same time, it can also be used for educational purposes, i.e., a virtual assistant to train novice colposcopists as what expert colposcopists would do during fellowship training. The detailed diagnostic workflow is shown in Fig. 1.

### The advancements in computer algorithms applying to cervical images

In recent years, we have witnessed the rapid development of deep learning techniques, which have been widely used for medical imaging and achieved impressive performances in various tasks such as lung nodule detection in CT and pathological image classification [16, 17]. At present, great progress has been made in developing AI-guided digital colposcopy to improve the efficiency and accuracy of clinical diagnosis. Some pilot studies in computer algorithms applying to cervical images were reported by some scholars (see Table 1).

**Table 1:** The advancements in computer algorithms applying to cervical images

Reference	Publish year	Aim of the study	Study design	Number of subjects	Image-generating devices	Type of algorithms	Outcomes
Simoes et al. [18]	2014	Classification of colposcopy images	Retrospective	170 images (training set 48; test and internal validation set 122)	Digital colposcopy	ANN	Accuracy 72.15%
Kim and Huang [19]	2013	Detection of CIN2+ from normal/CIN1	Retrospective	2000 images (normal/CIN2 1000; CIN2+ 1000)	Cervicography (discontinued)	SVM	Sensitivity 75% Specificity 75%
Asiedu et al. [20]	2019	Detection of CIN1+ against normal	Retrospective	134 patients (training set 107; internal validation set 27)	Digital colposcopy	SVM	Accuracy 80% Sensitivity 81.3% Specificity 78.6%
Miyagi et al. [21]	2019	Classification of CIN1 and CIN2+	Retrospective	310 images (both using in training and internal validation set)	Traditional colposcopy	Convolutional neural networks	Accuracy 82.3% Sensitivity 80% Specificity 88.2%
Song et al. [22]	2015	Detection of CIN2+	Retrospective	7669 patients with < CIN2, 142 patients with CIN2+ (training set 7531; internal validation set 280)	Cervicography (discontinued)	Multimodal convolutional neural networks	Accuracy 89% Sensitivity 83.21% Specificity 94.79%
Schiffman et al. [23]	2019	Detection of CIN2+	Retrospective	9127 patients with < CIN2, 279 patients with CIN2+ (training set 744, internal validation set 324, rest in screening set)	Cervicography (discontinued)	Faster R-CNN	AUC 0.91

Simoes et al. explored artificial neural networks with 170 colposcopy images as a training and validation set, and the results showed an accuracy of 72.15% [18]. Kim et al. trained a support vector machine (SVM) with 2000 cervical images and achieved the sensitivity and specificity of 75% for CIN2+ and normal/CIN1 classification [19]. Asiedu et al. trained and validated a SVM model with 134 patients and achieved 80% accuracy, 81.3% sensitivity, and 78.6% specificity for detecting CIN1+ against normal/benign tissues [20]. Miyagi et al. trained and validated AI classifier by using 310 images, which showed a high accuracy of 82.3%, sensitivity of 80%, and specificity of 88.2% for the classification of CIN1 and CIN2+ [21]. Song et al. trained multimodal convolutional neural networks with 60,000 colposcopy images and related clinical records (age, pap tests, and HPV information), which showed a high accuracy of 89%, sensitivity of 83.21%, and specificity of 94.79% for CIN2+ detection [22]. In addition, Schiffman et al. reported an AUC of 0·91 achieved by Faster region-based convolutional neural network (Faster R-CNN) in detecting precancers using limited 279 CIN2+ cases [23]. These studies indicate an encouraging trajectory, but they could not be generalized due to the relatively small training set and lack of external validations/prospective clinical trials to confirm the results in clinical settings. In the future, we expect more medical evidence to unlock the enigma of AI-guided digital colposcopy.

### The challenges to development and application of AI-guided digital colposcopy

Despite the promising performance of AI with colposcopy imaging, several challenges and obstacles remain for further development and application.

Firstly, with the increasing demand for colposcopy imaging in LMICs, large-scale datasets of colposcopic images are generated by hospitals. However, these datasets are rarely well managed for labeling, annotation, classification, and quality control due to different types of colposcopy equipment used for data collection and non-uniform descriptive terminology in colposcopy practice, which makes it difficult for the data to be used for training and validating AI-guided digital colposcopy. The management of medical data is one of the main challenges for the development of AI models. The ultimate solution is to standardize colposcopy equipment and terminology; however, it is difficult for this alignment in a short time. Another reasonable strategy is to further improve the AI algorithms to enable them to deal with images of different origins and qualities.

Secondly, in clinical practice, the dynamic changes of appearance of acetowhitening epithelium after the application of an acetic acid solution of 3–5% are viewed as the main diagnostic basis for colposcopists [24]. However, most existing studies only applied static colposcopy images rather than real cervical regions to develop AI models, resulting in information bias in feature extraction of cervical lesions. This could in turn affect the diagnostic accuracy of AI models. Also, how to embed the AI models in the existing colposcopy imaging equipment becomes the challenge for AI analysis of dynamic imaging.

Thirdly, the current AI models lack the interpretation of diagnostic decisions. The black boxes of AI models decrease their convincingness to colposcopists. In fact, interpretability and explainability of AI models are necessary to assist clinical colposcopists and provide relevant recommendations, such as the severity of the cervical lesions, biopsy sites, and even specific diagnostic evidence. This will make it easier for colposcopists to accept these AI models in clinical practice and could even become a teaching tool in hospitals or medical schools.

Last but not least, using AI models to diagnose a certain rare cervical disease or complex complications is still unreliable as a number of presentations are rare in clinical practice, which is usually insufficient for the training and validation of AI models. Thus, the concept of developing and applying AI models in clinical practice is to help colposcopists improve the diagnostic ability rather than replace them. The diagnostic results of the AI model are only used as a reference, and human colposcopists should continue to be responsible for the final result.

### The suggestions for implementation of AI-guided digital colposcopy

#### Data quality control and data security

To develop an effective and robust AI-guided digital colposcopy, we must select high-quality colposcopy images and quality-controlled pathology diagnoses as the training set to ensure its diagnostic accuracy and reliability. The structure of learning data is very complex, particularly in LMICs with an extremely imbalanced medical resource; thus, this leads to extensive information islands. It is necessary for data professionals to closely work with colposcopists to deal with raw data for image annotation, classification, and quality control. Moreover, due to the requirement of a large amount of data to develop an AI model, the risk of data privacy disclosure and data sharing becomes an important concern. It is necessary to enhance data management and system authority, desensitize data, and implement a comprehensive privacy standard to reduce the risk of personal privacy disclosure.

#### Target positioning of AI-guided digital colposcopy

The AI-guided digital colposcopy aims to assist colposcopists rather than replace them. Also, colposcopists should not rely on AI-guided digital colposcopy, especially for junior colposcopists. Otherwise, junior colposcopists may still have the risk of being replaced. Meanwhile, colposcopists should continuously improve their ability from AI-assisted model and find the problems in AI interpretation, rather than simply apply the AI-assisted technology. Admittedly, the combination of colposcopists and AI model could improve the diagnostic ability of colposcopy, augment clinical judgment, and eventually compensate for the lack of experienced colposcopy practitioners in LMICs. Although the AI model can make a correct image-based subjective diagnosis in some aspects, colposcopists often consider other cervical disease-related factors (e.g., age, primary screening results, self-reported symptoms, or clinical features of suspected cervical diseases) and are better at integrating information to make decisions than machines. Currently, AI products play an auxiliary role in the field of medicine, for example, cancer screening, diagnosis, medical imaging recognition, disease rehabilitation, and treatment. But with time, the application will continue to expand as the AI models are being adapted to integrate and digest multimodality datasets. In addition, the function positioning remains unclear for the development and application of AI-guided digital colposcopy. At present, there are two main available choices of target development that have been used to classify colposcopy images. Some researches prefer to define the outcome based upon the colposcopic impression of high-grade or worse threshold for treatment. Others base the outcome on the low-grade or worse threshold to guide cervical biopsies for reducing missed CIN2+ diagnosis as much as possible, which is the current colposcopic bottleneck limiting the screening performance in LMICs. We urgently need a practice AI-guided digital colposcopy that assists colposcopists to guide cervical biopsy precisely as the top priority to improve diagnostic performance.

#### Team building of AI-guided digital colposcopy

The AI-guided digital colposcopy can be developed and applied by building an interdisciplinary team of epidemiologists, colposcopists, pathologists, AI engineers, and product engineers. The epidemiologists in the whole process should navigate the study design, provide decision support, and promote research and development. Also, when colposcopists can find the problems existing in the current AI-assisted model in clinical application, the frequent communication between colposcopists and AI engineers can promote the development of AI-guided digital colposcopy. This multidisciplinary integration from different professional talents will encourage and promote close cooperation between universities, cancer research institutes, hospitals, and medical enterprises to establish a new discipline. Finally, this innovative discipline will attract more advanced talents to implement innovations in the field of AI, drive the development of interdisciplinary subjects, and promote AI algorithm transformation into clinical application.

#### Development of AI regulations and laws

Currently, there are deficient laws and regulations at the national level to regulate the development and application of medical AI technology, accountability sharing mechanisms, and supervision of data and privacy in most LMICs [25]. The deficiencies bring the risk of unclear subject responsibility in AI applications, which hinders innovation and industrial development of medical AI. Therefore, it is necessary to provide appropriate legal regulation and ethical guidance for medical artificial intelligence from the aspects of clarifying legal subjects, clearly dividing legal responsibilities, protecting data and privacy, and establishing social norms and ethical standards for the better application of medical AI products to promote human health.

## Conclusion

Colposcopy is rapidly transitioning into the digital era with substantial potential impact on developing AI. The combination of digital colposcopy and AI could bring exciting changes to the prevention of cervical cancer, although plenty of technical, ethical, and legal issues remain to be solved. AI-guided digital colposcopy could assist colposcopists to improve their diagnostic performance, optimize clinical workflow, and mitigate the pressure of colposcopists and hospitals. The construction of cloud platform-based AI-guided digital colposcopy could create a novel cervical cancer screening model and provide equal access to diagnostic tools for cervical cancer in any regions of LMICs. We hope all research institutes or universities should translate their AI algorithms into clinical application and public health service, which thereby boosts AI-guided digital colposcopy to enter the health market and accelerates the global target of elimination of cervical cancer.

## Data Availability

Not applicable.

## Abbreviations

AI: Artificial intelligence
WHO: World Health Organization
LMICs: Low- and middle-income countries
CIN: Cervical intraepithelial neoplasia
HPV: Human papillomavirus
SVM: Support vector machine
